# Narrowing the localization of the region breakpoint in most frequent Robertsonian translocations

**DOI:** 10.1007/s10577-014-9439-3

**Published:** 2014-09-02

**Authors:** Malgorzata Jarmuz-Szymczak, Joanna Janiszewska, Krzysztof Szyfter, Lisa G. Shaffer

**Affiliations:** 1 Institute of Human Genetics, Polish Academy of Sciences, ul. Strzeszynska 32, 60-479 Poznan, Poland; 2 Department of Hematology, Poznan University of Medical Sciences, Poznan, 60-569 Poland; 3 Paw Print Genetics, Genetic Veterinary Sciences, Inc, Spokane, WA 99202 USA

**Keywords:** Robertsonian translocation, BAC clones, breakpoint, Translocation formation, Acrocentric chromosome

## Abstract

Despite that Robertsonian translocations (ROBs) are the most common chromosomal rearrangements in humans (1/1000 individuals), an exact breakpoint and the molecular mechanisms leading to their formation are still not well known. This is partly due to the fact that Human Genome Project did not provide any map or sequence for the acrocentric short arms. The main aim of our studies was to narrow the breakpoints in de novo arising and in familial cases of the most frequently occurring ROBs, using eight, previously not tested clones derived from 21p. Our results from PCR and FISH analysis showed that only the clones CR382285, CR382287, and a small fragment of CR382332 are retained in the examined ROBs. Moreover, interphase FISH on monochromosomal hybrids verified the orientation of studied clones in relation to centromeres of chromosomes 14 and 21. Given our results, we propose localization of the breakpoints in or nearby to clone CR382332. Summarizing, our results allowed to narrow the region where the breakpoints are localized and demonstrated that their position could be the same in all common ROBs.

**Electronic supplementary material:**

The online version of this article (doi:10.1007/s10577-014-9439-3) contains supplementary material, which is available to authorized users.

## Introduction

Robertsonian translocations (ROBs) are the most common chromosomal rearrangements in humans, with an incidence of approximately 1/1000 individuals (Therman et al. 1989). They arise through exchanges between the short arms of acrocentric chromosomes: 13, 14, 15, 21, and 22. Exchanges within these five chromosomes may form ten nonhomologous Robertsonian translocations, but their distribution is highly nonrandom with the predominance of rob(13;14) (75 %) and rob(14;21) (10 %) (Therman et al. 1989). These two, most common Robertsonian translocations (mcROBs) are the subject of this work.

The molecular mechanisms leading to mcROBs formation are still not well known, except the observation that they mainly arise during oogenesis (Shaffer 2002). It is possible that sequences found on chromosomes 13, 14, and 21 mediate homologous recombination resulting in these translocations. Moreover, it was postulated that the order of sequences in 14p are in opposite orientation than topography of homologous sequences in 13p and 21p (Therman et al. 1989; Choo et al. 1988; Shaffer 2002).

**Fig. 1:**
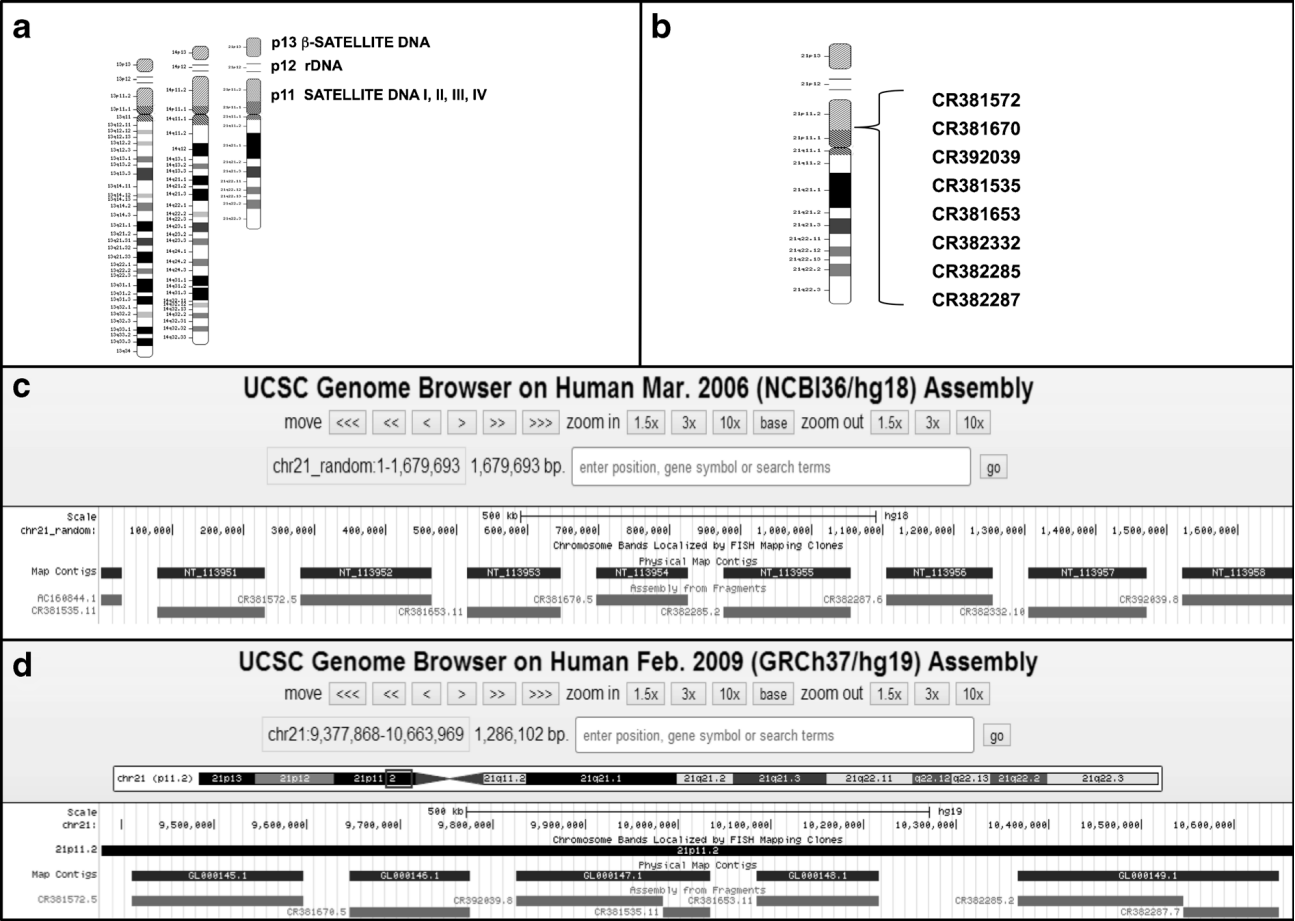
**a** The collocation of analogous sequences in short arm of acrocentric chromosomes. **b** The order of eight BAC clones in 21p described by Lyle et. al. **c** The first occurrence of eight examined BAC clones in UCSC GENOME BROWSER database assigned as random in chromosomes 21. **d** The latest version of UCSC GENOME BROWSER database, showing the exact localization of seven out of eight BAC clones (except CR382332) in chromosome 21

Additionally, the exact breakpoints in mcROBs are not fully recognized because the short arms of acrocentric chromosomes are still poorly understood regions of the genome. This is due to the fact that in the Human Genome Project, only coding sequences were mapped, while noncoding, heterochromatic regions, including the short arms of chromosomes 13, 14, 15, 21, and 22, were omitted. Even though, it is known that the p arms of these chromosomes are divided between three bands: (i) p11, which contains satellite I-IV (Choo et al. 1990; Choo et al. 1992; Gosden et al. 1981; Gravholt et al. 1992) and β-satellite sequences (Waye and Willard 1989); (ii) p12 containing the 18S and 28S ribosomal genes (Worton et al. 1988); and (iii) p13 with β-satellite DNA (Waye and Willard 1989) (Fig. 1a). Some other subfamilies of satellite DNA specific to these regions have been described (Kalitsis et al. 1993) (Choo et al. 1992), (Choo et al. 1990); (Bandyopadhyay et al. 2001b).

The known sequences of satellite DNA have been used to determine the regions containing the breakpoints in ROB. To date, it has been established that in mcROBs, rRNA genes located in band p12 undergo deletions in all involved chromosomes (Gosden et al. 1979; Gravholt et al. 1992; Han et al. 1994; Mattei et al. 1979; Wolff and Schwartz 1992). Moreover, there are some indications that the breakpoint might be located (i) in 14p between pTRS-47 subfamily—adjacent to the centromere (present in 97 % of the analyzed mcROB) and pTRS-63, which is more distal (deleted in mcROBs) (Earle et al. 1992) or (ii) in chromosomes 13 and 21 between the satellite I DNA pTRI-6, and the rRNA genes (Kalitsis et al. 1993). Furthermore, it was also found that subfamilies of satellite III DNA: pTRS-63 and pR-2 in some patients and pE-1 and pR-4 in all rob(14;21) are deleted, while the sequences of pTRS-47, pK-1, pE-2, and pR-1 are retained (Bandyopadhyay et al. 2001a).

In 2007, Lyle et al. have developed genome map of acrocentric chromosomes by sequencing of eight new BAC clones (CR382285, CR382287, CR381572, CR381535, CR381653, CR382332, CR381570, CR392039) which were identified by STSs from the CHORI-507 library (Lyle et al. 2007) and localized in the p arm of chromosome 21 (Fig. 1b). In 2006, the sequences of these clones were placed randomly on chromosome 21 in the Genome Browser database (GRch36/hg18) (Fig. 1c); in 2009, they were assigned to 21p11 (Genome Browser and NCBI- GRch37/hg19) (Fig. 1d, Table S1). Despite that these clones were described in 2007 and provided opportunity to continue studies on acrocentric chromosomes, up to now, nobody has studied their occurrence and configuration in ROBs.

In order to narrow the region of breakpoints, we decided to use these eight clones from 21p to verify their presence and location in 20 patients with rob(13;14) or rob(14;21), which have arisen either *de novo* or have been familial.

## Materials and methods

### Cell lines

The material consisted of the following: (i) 14 lymphoblastic cell lines and 6 mouse-human or hamster-human hybrids derived from patients with mcROBs: rob(14;21) or rob(13;14), (ii) 6 control lymphoblastic cell lines from normal male and female, (iii) somatic monochromosomal hybrids, (iv) 6 lymphoblastic cell lines from patients’ mothers, (v) 2 hybrids with chromosomes from patients’ mothers, (vi) 2 lymphoblastic cell lines from Polish patients’ family members without translocation, and (vii) hamster and mouse fibroblast cell lines. Cell line characteristics and culture conditions are described in Table S2.

Altogether, we analyzed six rob(14;21) arising de novo and one of unknown origin as well as thirteen rob(13;14) including the following: de novo (4 cases), paternally inherited (6 cases), maternally inherited (2 cases), and unknown origin (1 case). De novo cases were derived from our long-term collection, while cases with familial occurrence were originated from one Polish family.

### Construction of somatic cell hybrids

Somatic cell hybrids were constructed from patient cell line, through its fusion with the HPRT-deficient hamster-derived cell line RJK88 or mouse cell line A9, using polyethylene glycol (PEG) (Zoghbi et al. 1989). The isolated colonies were screened by PCR with polymorphic microsatellite markers, mapping to the long arm of each acrocentric chromosome (D13S1275, D13S162, D13S175, D14S128, D14S139, D14S283, D21S1276, D21S188), to identify the hybrid that retained the chromosomes 13 and 14 or 14 and 21. FISH analysis with probes for centromere regions of chromosomes 13 and 21 (D21Z1/D13Z1), and 14 and 22 (D14Z1/D22Z1) was used to distinguish between the hybrids containing the following: (i) only the translocation, (ii) free-lying homologous chromosomes or, (iii) in some cases, both the translocation and one or more free-lying chromosomes. Translocations were also determined to be monocentric or dicentric using the same centromeric FISH probes.

### FISH analysis

Standard procedure was used to prepare metaphases from 20 cell lines with mcROBs, 11 controls with chromosomes from family members, and 6 normal controls (3 male and 3 female). Controls served as the basis for exclusion of polymorphisms and verification of probes localization.

At the beginning, the FISH analyses on mcROB cases were performed with alpha satellite probes D21Z1/D13Z1, D14Z1/D22Z1 (Kreatech). Then, FISH was done on mcROBs as well as on controls using probes prepared from the eight BAC clones: CR382285, CR382287, CR381572, CR381535, CR381653, CR382332, CR381670, and CR392039. DNA from BAC clones were biotin or digoxigenin-labeled by nick translation. Additionally, clones CR382285, CR381572, and CR382332 were used in interphase FISH on monochromosomal hybrids containing chromosome 13, 14, or 21 according to Gajecka et al. (Gajecka et al. 2005). These clones were hybridized to interphases in the presence of alpha satellite probes, specific to centromere region of chromosomes involved in mcROBs in two combinations: (1) CR381572, CR382332, and alpha satellite probes and (2) CR381572, CR382285, and alpha satellite probes. The FISH analyses, both on lymphoblastic cell lines and hybrids, were conducted using standard procedures (Shaffer et al. 1994).

### DNA extraction and PCR analysis

DNA from cell lines was isolated using proteinase K and phenol-chloroform extraction. Primers, based on sequences derived from eight BAC clones, were designed using Primers 3 software (http://bioinfo.ut.ee/primer3-0.4.0/). PCR reactions were performed using *Taq* DNA polymerase (Fermentas). Most primers had various PCR conditions with the main differences concerning the annealing time (45–90 s) and the annealing temperature (Table S3). The clones and primers are shown in Table 1 and Table S1, respectively.

**Table 1:** FISH results with BAC clones for controls. Listed chromosomes, where signals are present mainly in pericentromeric regions

	Controls					
BAC clones	Male 1	Male 2	Male 3	Female 1	Female 2	Female 3
CR382285 (bP-21201H5)	4q28, 9, 13, 14, 21	4q28, 9, 13, 14, 21	4q28, 9, 13, 14, 21	4q28, 9, 13, 14(1), 21	4q28, 9, 13, 14, 21	4q28, 9, 13, 14, 21
CR381572 (bP-2168N6)	1, 2, 7, 9, 10, 13–15, 16, 21, 22	2, 7, 9, 10, 13–15, 16, 21, 22	1, 2, 7, 9, 10w, 13–15, 16, 21, 22	1, 2, 7, 9, 10, 13, 14, 15, 16, 21, 22,	1, 2, 7, 9, 10, 13–15, 16, 21, 22	2, 7, 9, 10, 13–15, 16, 21, 22
CR381535 (bP-2154M18)	13–15, 21, 22	4, 9q, 13–15, 21, 22	4, 9q, 13–15, 21, 22	1, 9q, 13–15, 21, 22	1, 9q, 13–15, 21, 22	1(1), 4(1), 9w, 13–15, 21, 22
CR381670 (bP-2189O9)	1, 4, 13–15, 21, 22	1, 4, 13–15, 21, 22	1, 9, 4, 13–15, 21, 22	1, 2, 3, 4, 9, 13–15, 18, 21, 22	1, 4, 9, 13–15, 20, 21, 22	1w, 4, 13–15, 21, 22
CR392039 (bP-2171C21)	Y, 9, 13–15, 21, 22	4, 9, 13–15, 21, 22	Y, 9, 13–15, 21, 22	9, 13–15, 21, 22	4, 9, 13–15, 21, 22	4(1), 9, 13–15, 21, 22
CR381653 (bP-21264C1)	1, 4, 13–15, 21, 22	1, 4, 13–15, 21, 22	1, 4, 13–15, 21, 22	1, 2, 3, 4, 9, 13–15, 18, 21, 22	1, 4, 13–15, 21, 22	1, 4, 13–15, 21, 22
CR382332^a^ (bP-21120F14)	1, 4, 9, 13–15, 21, 22	1, 4, 9w, 13–15, 21, 22	1, 4, 9w, 13–15, 21, 22	9, 13–15, 21, 22	1, 4, 9, 13–15, 21, 22	1, 4, 9w, 13–15, 21, 22
CR382287 (bP-21216K13)	4q28, *9* **,** *14*, 13, 21, 22,	4q28, 9, 13–15, 21, 22	4q28, 9, 13–15, 21, 22	4q28, 9, 13, 14, 21, 22, 15(1)	4q28, 9, 13, 14, 21, 22, 15(1)	4q28, 9, 13–15, 21, 22

Very strong signals are in italic

*w* weak signal, *(1)* means that hybridization signal was observed only on one chromosome

^a^Working draft sequence

## Results

### FISH analysis

FISH analyses using alpha satellite probes D21Z1/D13Z1 and D14Z1/D22Z1 have revealed the dicentric nature of all studied mcROBs (data not shown). These outcomes for the de novo cases were previously published (Han et al. 1994).

**Fig. 2:**
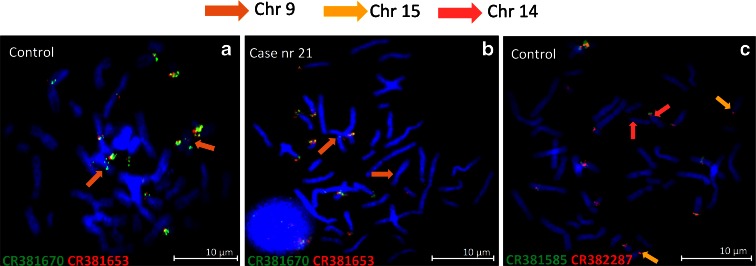
The FISH results present the differences in the hybridization pattern between individuals: presence of clone CR381670 on chromosome nine in one male control (**a**), absence of clone CR381670 in second (**b**). The occurrence polymorphism of clones CR382285 and CR382287 (**c**)

The results with eight studied BAC clones on normal metaphases derived from three males and three females are shown in Table 1 and Fig. 2.

**Fig. 3:**
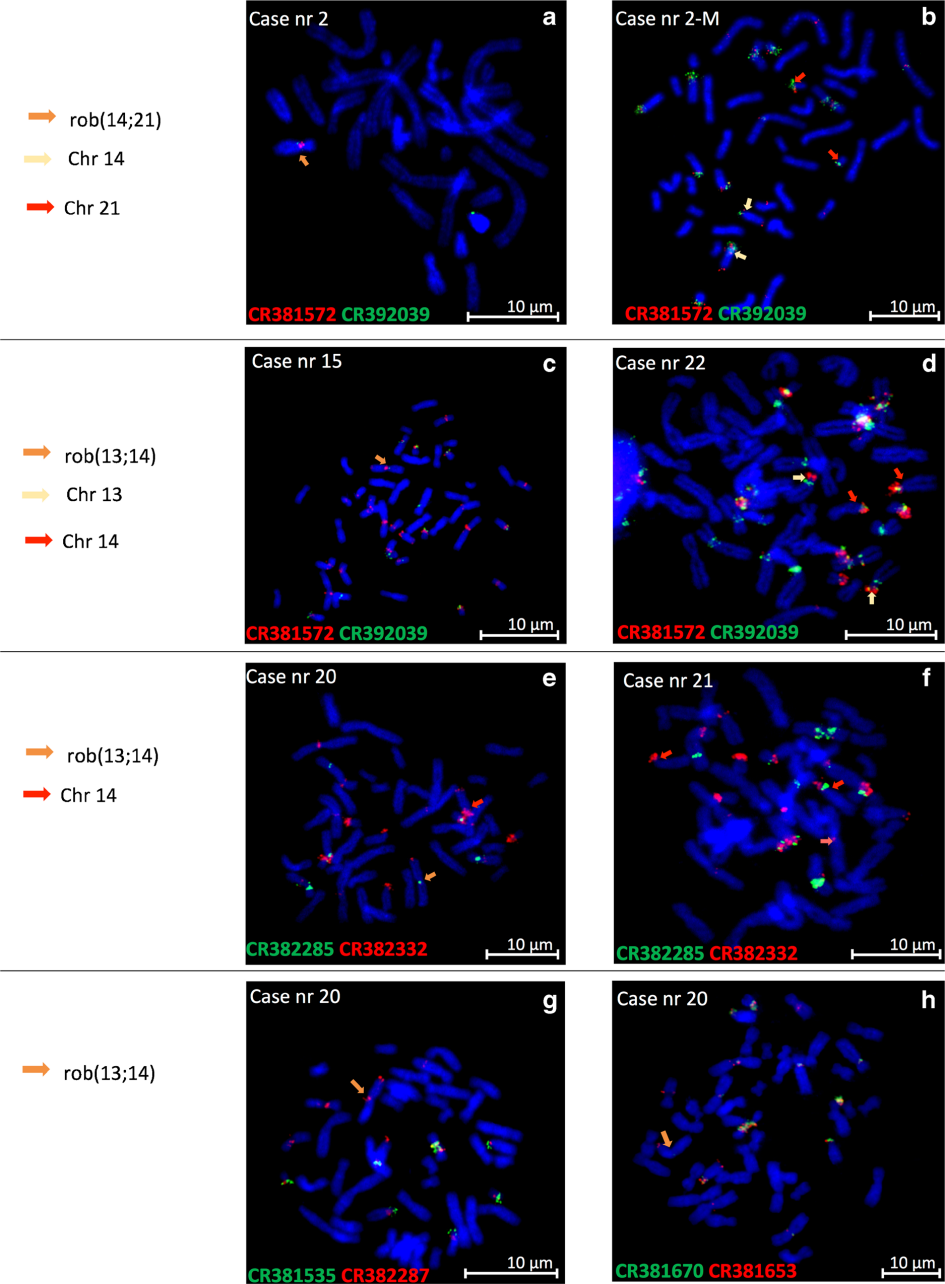
The FISH results on mcROBs {(**a**) rob(14;21) and (**c** and **f**) rob(13;14)} and mcROB’s mother (**b**) and family member (**d** and **e**) demonstrate presence of analyzed BAC clones on acrocentric chromosomes involved in translocation formation. The results of FISH analysis: case 20 with CR382287 and CR381535 (**g**), and with CR381670 and CR381653 (**h**). A red telomere probe for chromosome 14

The metaphase FISH on rob(13;14) or rob(14;21) indicates that only clones CR382285, CR382287, and CR381572 are present in Robertsonian translocations (Fig. 3). The same time, clones CR381535, CR381653, and CR382332 were absent (Fig. 3). Very weak hybridization signals for clones CR381670 and CR392039 were present (Table 2), which could be explained by cross hybridization with homologous sequences. Whereas FISH results on mcROB’s mothers and family members demonstrate presence of all analyzed BAC clones on acrocentric chromosomes involved in translocation formation (Fig. 3).

**Table 2:** FISH results with BAC clones for ROB

Case	Sample Type	Origin	Karyotype	CR382287	CR382285	CR382332*	CR381653	CR381535	CR392039	CR381670	CR381572
1	somatic hybrid	de novo	rob(14;21)	+	+	−	−	−	+w	+w	+
2	somatic hybrid	de novo	rob(14;21),4	+	+	−	−	−	+w	+w	+
3	somatic hybrid	de novo	rob(14;21),Y	+	+	−	−	−	+w	+w	+
4	somatic hybrid	de novo	rob(14;21),9	+	+	−	−	−	+w	+w	+
5	somatic hybrid	de novo	rob(14;21),9q	+	+	−	−	−	+w	+w	+
6	lymphoblast	de novo	45,XX,rob(14;21)	+	+	−	−	−	+w	+w	+
7	lymphoblast	unknown	46,XY,rob(14;21),+21	+	+	−	−	−	+w	+w	+
8	somatic hybrid	de novo	rob(13;14), 13	+	+	−	−	−	+w	+w	+
9	lymphoblast	de novo	45,XX,rob(13;14)	+	+	−	−	−	+w	+w	+
10	lymphoblast	de novo	45,XX,rob(13;14)	+	+	−	−	−	+w	+w	+
11	lymphoblast	de novo	45,XY,rob(13;14)	+	+	−	−	−	+w	+w	+
12	lymphoblast	paternal	45,XX,rob(13;14)	+	+	−	−	−	+w	+w	+
13	lymphoblast	paternal	45,XX,rob(13;14)	+	+	−	−	−	+w	+w	+
14	lymphoblast	paternal	45,XX,rob(13;14)	+	+	−	−	−	+w	+w	+
15	lymphoblast	maternal	45,XY,rob(13;14)	+	+	−	−	−	+w	+w	+
16	lymphoblast	unknown	45,XY,rob(13;14)	+	+	−	−	−	+w	+w	+
17	lymphoblast	paternal	45,XY,rob(13;14)	+	+	−	−	−	+w	+w	+
18	lymphoblast	maternal	45,XY,rob(13;14)	+	+	−	−	−	+w	+w	+
19	lymphoblast	paternal	45,XX,rob(13;14)	+	+	−	−	−	+w	+w	+
20	lymphoblast	paternal	45,XX,rob(13;14)	+	+	−	−	−	+w	+w	+

+ signals are present, − signal is absent, +w the weak signal (clones order according to Lyle et al. 2007)

**Fig. 4:**
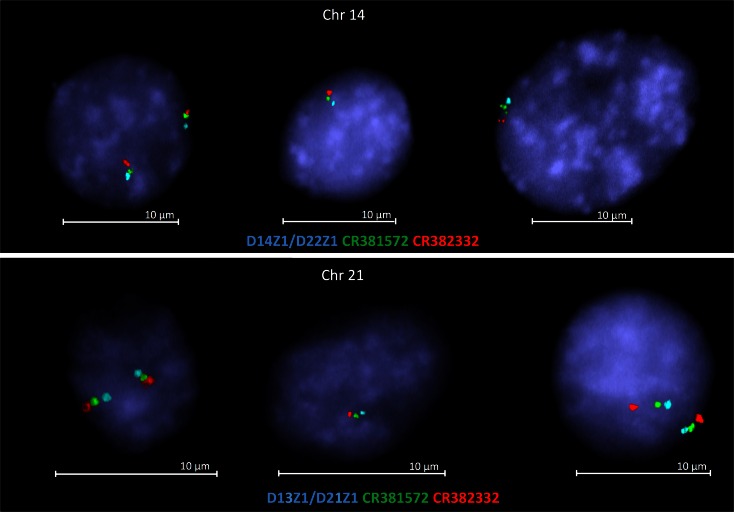
The interphase FISH results show centromere-telomere orientation of BAC clones CR381572 and CR382332 on 14p and 21p

Additionally, the interphase FISH results determined the centromere-telomere orientation of BAC clones CR381572 and CR382332 on 14p and 21p as follows: centromere, CR381572, CR382332, and telomere (Fig. 4). Unfortunately, this analysis did not give any unambiguous answer about orientation of CR382285 in relation to CR381572 and centromeres of chromosomes 13, 14, and 21.

### PCR results

In order to confirm FISH results, PCR analyses were conducted. Because of polymorphic character of analyzed sequences, only DNA from four somatic hybrids containing rob(14;21) were used. DNA from patients could give false positive results due to content of remaining chromosomes. DNA from monochromosomal hybrids with normal human chromosome 13−15, 21, 22, 4, 9, Y, mouse cell line A9, hamster cell line RJK88, and hybrids with chromosomes from mothers of children with de novo translocations (1-Ma and 1-Mb) were applied as controls. The molecular analysis demonstrated occurrence of PCR products for clones CR382285, CR382287, CR381572, and a small fragment of CR382332 in all somatic cell hybrids containing mcROB (cases 1, 2, 3, and 4). Additionally, PCR did not amplify products for clones CR381535, CR381653, CR381670, and CR392039 in one hybrid containing rob(14;21) as the only human acrocentric chromosome (hybrid 1, Table 3).

**Table 3:** The results of PCR with primers specific for the analyzed clones for all examined probes

Clones	Primers	Control DNA		Monochromosomal hybrid cell lines												Somatic hybrid cell lines with					
																Chromosomes from mothers		rob(14;21)			
		RJK88 (Hamster)	A9 (Mouse)	13 (GM10898D)	14 (GM11535A)	14 (GM10479B)	14 (CP43)	15 (GM 11715A)	21 (GM 10323A)	21 (GM08854)	22 (GM 10888A)	1 (GM 13139A)	Y (GM 63171A)	4 (GM 10115B)	9 (GM 10611)	1 Ma	1 Mb	1	2	3	4
CR382287	1	**−**	**−**	**+**	**+**	**+**	**+**	**−**	**+**	**+**	**−**	**−**	**−**	**+**	**+**	**+**	**+**	**+**	**+**	**+**	**+**
	2	**−**	**−**	**+**	**+**	**+**	**+**	**+**	**+**	**+**	**−**	**−**	**−**	**+**	**+**	**+**	**+**	**+**	**+**	**+**	**+**
	3	**−**	**−**	**+**	**+**	**+**	**+**	**+**	**+**	**+**	**−**	**−**	**−**	**+**	**+**	**+**	**+**	**+**	**+**	**+**	**+**
	4	**−**	**−**	**+**	**+**	**+**	**+**	**−**	**+**	**+**	**−**	**−**	**−**	**+**	**+**	**+**	**+**	**+**	**+**	**+**	**+**
	5	**−**	**−**	**+**	**+**	**+**	**+**	**−**	**+**	**+**	**−**	**−**	**−**	**+**	**+**	**+**	**+**	**+**	**+**	**+**	**+**
	6	**−**	**−**	**+**	**+**	**+**	**+**	**−**	**+**	**+**	**−**	**−**	**−**	**+**	**+**	**+**	**+**	**+**	**+**	**+**	**+**
	7	**−**	**−**	**+**	**+**	**+**	**+**	**+**	**+**	**+**	**−**	**−**	**−**	**+**	**+**	**+**	**+**	**+**	**+**	**+**	**+**
CR382285	1	**−**	**−**	**+**	**+**	**+**	**+**	**−**	**+**	**+**	**−**	**−**	**−**	**+**	**+**	**+**	**+**	**+**	**+**	**+**	**+**
	2	**−**	**−**	**+**	**+**	**+**	**+**	**−**	**+**	**+**	**−**	**−**	**−**	**+**	**+**	**+**	**+**	**+**	**+**	**+**	**+**
	3	**−**	**−**	**+**	**+**	**+**	**+**	**−**	**+**	**+**	**−**	**−**	**−**	**+**	**+**	**+**	**+**	**+**	**+**	**+**	**+**
	4	**−**	**−**	**+**	**+**	**+**	**+**	**−**	**+**	**+**	**−**	**−**	**−**	**+**	**+**	**+**	**+**	**+**	**+**	**+**	**+**
	5	**−**	**−**	**+**	**+**	**+**	**+**	**−**	**+**	**+**	**−**	**−**	**−**	**+**	**+**	**+**	**+**	**+**	**+**	**+**	**+**
	6	**−**	**−**	**+**	**+**	**+**	**+**	**−**	**+**	**+**	**−**	**−**	**−**	**+**	**+**	**+**	**+**	**+**	**+**	**+**	**+**
	7	**−**	**−**	**+**	**+**	**+**	**+**	**−**	**+**	**+**	**−**	**−**	**+**	**+**	**+**	**+**	**+**	**+**	**+**	**+**	**+**
CR382332	9	**−**	**−**	**+**	**+**	**+**	**+**	**+**	**+**	**+**	**+**	**+**	**+**	**+**	**+**	**+**	**+**	**+**	**+**	**+**	**+**
	8	**−**	**−**	**+**	**+**	**+**	**+**	**−**	**+**	**+**	**+**	**+**	**+**	**+**	**+**	**+**	**+**	**+**	**+**	**+**	**+**
	7	**−**	**−**	**+**	**+**	**+**	**+**	**+**	**+**	**+**	**+**	**+**	**+**	**+**	**+**	**+**	**+**	**+**	**+**	**+**	**+**
	13	**−**	**−**	**+**	**+**	**+**	**+**	**+**	**+**	**+**	**+**	**+**	**+**	**+**	**+**	**+**	**+**	**+**	**+**	**+**	**+**
	14	**−**	**−**	**+**	**+**	**−**	**+**	**+**	**+**	**+**	**+**	**−**	**+**	**+**	**−**	**+**	**+**	**−**	**+**	**−**	**+**
	15	**−**	**−**	**+**	**+**	**+**	**+**	**+**	**+**	**+**	**+**	**−**	**+**	**−**	**−**	**+**	**+**	**−**	**+**	**−**	**+**
	10	**−**	**−**	**+**	**+**	**+**	**+**	**+**	**+**	**+**	**+**	**−**	**+**	**+**	**−**	**+**	**+**	**−**	**+**	**−**	**+**
	4	**−**	**−**	**+**	**+**	**+**	**+**	**+**	**+**	**+**	**+**	**−**	**+**	**+**	**+**	**+**	**+**	**−**	**+**	**−**	**+**
	12	**−**	**−**	**+**	**+**	**+**	**+**	**+**	**+**	**+**	**+**	**+**	**−**	**+**	**+**	**+**	**+**	**+**	**+**	**+**	**−**
	5	**−**	**−**	**+**	**+**	**+**	**+**	**+**	**+**	**+**	**+**	**−**	**+**	**+**	**+**	**+**	**+**	**−**	**+**	**−**	**+**
	6	**−**	**−**	**+**	**+**	**+**	**+**	**+**	**+**	**+**	**+**	**−**	**+**	**−**	**−**	**+**	**+**	**−**	**−**	**−**	**+**
	1	**−**	**−**	**+**	**+**	**+**	**+**	**+**	**+**	**+**	**+**	**−**	**+**	**+**	**+**	**+**	**+**	**−**	**+**	**+**	**+**
	2	**−**	**−**	**+**	**+**	**+**	**+**	**+**	**+**	**+**	**+**	**−**	**+**	**+**	**+**	**+**	**+**	**−**	**−**	**−**	**+**
	3	**−**	**−**	**+**	**+**	**+**	**+**	**+**	**+**	**+**	**+**	**−**	**+**	**+**	**+**	**+**	**+**	**−**	**−**	**−**	**+**
CR381653	1	**−**	**−**	**+**	**+**	**+**	**+**	**+**	**+**	**+**	**+**	**−**	**+**	**+**	**+**	**+**	**+**	**−**	**+**	**−**	**+**
	2	**−**	**−**	**+**	**+**	**+**	**+**	**+**	**+**	**+**	**+**	**−**	**−**	**+**	**−**	**+**	**+**	**−**	**+**	**−**	**+**
	1a	**−**	**−**	**+**	**+**	**+**	**+**	**+**	**+**	**+**	**+**	**−**	**−**	**−**	**−**	**+**	**+**	**+**	**+**	na	na
	2a	**−**	**−**	**+**	**+**	**+**	**+**	**+**	**+**	**+**	**+**	**−**	**−**	**−**	**−**	**+**	**+**	**−**	**+**	na	na
	3	**−**	**−**	**+**	**+**	**+**	**+**	**+**	**+**	**+**	**+**	**−**	**−**	**−**	**−**	**+**	**+**	**−**	**+**	na	na
	5	**−**	**−**	**+**	**+**	**+**	**+**	**+**	**+**	**+**	**+**	**+**	**+**	**+**	**+**	**+**	**+**	**−**	**+**	na	na
	7	**−**	**−**	**+**	**+**	**+**	**+**	**+**	**+**	**+**	**+**	**−**	**+**	**+**	**+**	**+**	**+**	**−**	**+**	na	na
	8	**−**	**−**	**+**	**+**	**+**	**+**	**+**	**+**	**+**	**+**	**+**	**+**	**+**	**+**	**+**	**+**	**−**	**+**	na	na
	10	**−**	**−**	**−**	**+**	**+**	**+**	**−**	**+**	**+**	**+**	**−**	**−**	**+**	**+**	**+**	**+**	**−**	**+**	na	na
CR381535	1	**−**	**−**	**+**	**+**	**+**	**+**	**+**	**+**	**+**	**+**	**−**	**−**	**−**	**+**	**+**	**+**	**−**	**−**	**−**	**+**
	2	**−**	**−**	**+**	**+**	**+**	**+**	**+**	**+**	**+**	**+**	**−**	**+**	**+**	**+**	**+**	**+**	**−**	**+**	**−**	**+**
	3	**−**	**−**	**−**	**+**	**+**	**+**	**+**	**+**	**+**	**+**	**−**	**+**	**+**	**+**	**+**	**+**	**−**	**+**	**−**	**+**
CR392039	1	**−**	**−**	**+**	**+**	**+**	**+**	**+**	**+**	**+**	**+**	**−**	**+**	**+**	**+**	**+**	**+**	**−**	**+**	**−**	**+**
	2	**−**	**−**	**+**	**+**	**+**	**+**	**+**	**+**	**+**	**+**	**−**	**+**	**−**	**−**	**+**	**+**	**−**	**+**	**−**	**+**
	3	**−**	**−**	**+**	**+**	**+**	**+**	**+**	**+**	**+**	**+**	**−**	**−**	**−**	**−**	**+**	**+**	**−**	**+**	**−**	**+**
CR381670	1	**−**	**−**	**+**	**+**	**+**	**+**	**+**	**+**	**+**	**+**	**−**	**+**	**+**	**+**	**+**	**+**	**−**	**+**	**−**	**+**
	2	**−**	**−**	**+**	**+**	**+**	**+**	**+**	**+**	**+**	**+**	**−**	**+**	**+**	**+**	**+**	**+**	**−**	**+**	**−**	**+**
	3	**−**	**−**	**+**	**+**	**+**	**+**	**+**	**+**	**+**	**+**	**−**	**+**	**+**	**+**	**+**	**+**	**−**	**+**	**−**	**+**
	4	**−**	**−**	**−**	**−**	**+**	**−**	**−**	**+**	**+**	**−**	**−**	**+**	**+**	**+**	**+**	**+**	**−**	**+**	**−**	**+**
	6	**−**	**−**	**+**	**+**	**+**	**+**	**+**	**+**	**+**	**+**	**−**	**+**	**+**	**+**	**+**	**+**	**−**	**+**	**+**	**+**
	7	**−**	**−**	**+**	**+**	**+**	**+**	**+**	**+**	**+**	**+**	**−**	**+**	**+**	**+**	**+**	**+**	**−**	**+**	**+**	**+**
	8	**−**	**−**	**+**	**+**	**+**	**+**	**+**	**+**	**+**	**+**	**−**	**+**	**+**	**+**	**+**	**+**	**+**	**+**	**+**	**+**
CR381572	1	**−**	**−**	**−**	**+**	**+**	**+**	**+**	**+**	**+**	**+**	**−**	**−**	**−**	**+**	**+**	**+**	**+**	**+**	**+**	**+**
	2	**−**	**−**	**−**	**+**	**+**	**+**	**+**	**+**	**+**	**+**	**−**	**−**	**−**	**+**	**+**	**+**	**+**	**+**	**+**	**+**
	3	**−**	**−**	**−**	**+**	**+**	**+**	**+**	**+**	**+**	**+**	**−**	**−**	**−**	**+**	**+**	**+**	**+**	**+**	**+**	**+**
	4	**−**	**−**	**+**	**+**	**+**	**+**	**+**	**+**	**+**	**+**	**+**	**+**	**−**	**+**	**+**	**+**	**+**	**+**	**+**	**+**
	5	**−**	**−**	**−**	**+**	**+**	**+**	**−**	**+**	**+**	**+**	**−**	**−**	**−**	**−**	**+**	**+**	**+**	**+**	**+**	**+**
	6	**−**	**−**	**+**	**+**	**+**	**+**	**+**	**+**	**+**	**+**	**+**	**+**	**−**	**+**	**+**	**+**	**+**	**+**	**+**	**+**

+ indicates the presence of the PCR product for each primer pair, − there is no product for a given pair of primers

*na* not analyzed (clones order according to Lyle et al. 2007)

## Discussion

Robertsonian translocations are the most common chromosomal rearrangements in humans, where short arms of acrocentric chromosomes are involved in their formation. Although the carriers of ROB have a normal phenotype, they very often have problems with unbalanced gametes formation through nondisjunction resulting in possibility of delivering chromosomally abnormal offspring. Despite that the ROB are very common, their molecular formation mechanism and breakpoint are still not well known.

The main aim of these studies was to narrow the region containing the breakpoints in de novo and in familial cases of the most frequently appearing rob(13;14) and rob(14;21). Our analysis was based on the BAC clones, first described by Lyle et al. who demonstrated the following order in 21p (from centromere to telomere): CR382287, CR382285, CR382332, CR381653, CR381535, CR392039, CR381670, and CR381572 (Lyle et al. 2007). Since 2009, the sequences of these clones (without CR382332) have been available in databases (UCSC Genome Bioinformatics, http://genome.ucsc.edu/; NCBI, http://www.ncbi.nlm.nih.gov/index.html) but their occurrence has been never studied in ROBs before.

Firstly in our studies, we verified the presence of these eight clones in normal male and female using FISH. Our results indicate that these sequences are present not only on short arm of chromosome 21 but also (i) on short arms of other acrocentric chromosomes, (ii) in pericentromeric regions of others chromosomes (1, 2, 3, 4, and 9). Additionally, in q arm of chromosome 4q28 two clones: CR382285 and CR382287 were found (Table 1). These findings are consistent with the results received in silico, which demonstrated a lot of duplications throughout the human genome (Jarmuz-Szymczak 2011; Lyle et al. 2007). However, FISH results presented by Lyle et al. showed less hybridization signals on human metaphases (Lyle et al. 2007) than were found in our study. It could be explained by stringency of FISH experiments and/or polymorphism of these sequences. In addition, we observed some differences in the hybridization pattern between individuals (Table 1). For instance, in one control, hybridization signals of CR382287 probe were stronger on chromosome 9 and 14p as compared to the remaining chromosomes, while in others, the signals were almost the same on each chromosome (Table 1). In addition, clone CR381670 demonstrated the polymorphism on chromosome 9 in two controls (Table 1, Fig. 2a–b). We also noticed the absence of clones CR382285 and CR382287 on one chromosome 14 and 15, respectively (Table 1, Fig. 2c). Because of these variances observed in healthy controls, we had to exclude polymorphisms in our patients. First of all, we verified whether the lack of clones CR382332, CR381535, CR381653, CR381670, and CR392039 observed in translocation carriers had not been the result of polymorphism in studied families. For this purpose, as the controls for the FISH analysis, metaphase chromosomes from mothers of de novo mcROB carriers as well as a male and a female with normal karyotypes from family segregating mcROB were used. The FISH results with all examined clones demonstrated the presence of all studied BAC clones on chromosomes participating in the family members of mcROBs (Fig. 3).

Due to exclusion of occurrence the polymorphism in studied cases and their families, we were able to indicate, based on FISH and PCR results, that clones CR382285, CR382287, and CR381572 are present in analyzed mcROB both arising de novo and in familial cases. Contrary to FISH results, the small fragment (about 2 kb) of CR382332 was detected by PCR in mcROBs. It may be explained by the size of this fragment, which is too small to be detected by FISH analysis. Results of both analyses for others clones demonstrated their absence in mcROBs. Even though we observed PCR product of clones CR381535, CR381653, CR381670, and CR392039 in three (cases 2, 3, 4) of four hybrids containing mcROB, we had to exclude presence of these clones in Robertsonian translocation because they were absent in case 1. The occurrence of PCR products in these three hybrids (2,3,4) is explained by the content of additional human chromosomes in which these sequences’ fragments are also present. It should be noticed that only the hybrid derived from case 1 did not contain other human chromosomes except rob(14;21) (Table 2).

The exact mechanism of Robertsonian translocation formation is still not known. However, it seems that the recombination between the repeated sequences of satellite III DNA or other repeated sequences occurring in the short arms of acrocentric chromosomes is probably involved. Moreover, factors such as a high frequency of de novo translocations formation in the population (Page and Shaffer 1997), non-random participation of certain acrocentric chromosomes in ROBs (Therman et al. 1989), and the formation of the majority of translocations during oogenesis (Page and Shaffer 1997) support the hypothesis that there must be a specific mechanism leading to the formation of rob(13;14) and rob(14; 21) (Page et al. 1996). Furthermore, it is very likely that sequences located on chromosomes 13, 14, and 21 that mediate homologous recombination eventually lead to the formation of these translocations (Choo et al. 1988; Therman et al. 1989). Consequently, it has been proposed (Choo et al. 1988; Shaffer 2002; Therman et al. 1989) that the chromosome 14 (i) shares homologous sequences with the short arms of chromosomes 13 and 21 and (ii) these sequences are arranged in the opposite orientation on chromosome 14 that has been proposed (Shaffer 2002). Moreover, these assumptions indicate that the formation of rob(13;14) and rob(14;21) is favored, but rob(13;21) is not, as assisted by the relatively low incidence of rob(13;21) in population (Shaffer 2002).

**Fig. 5:**
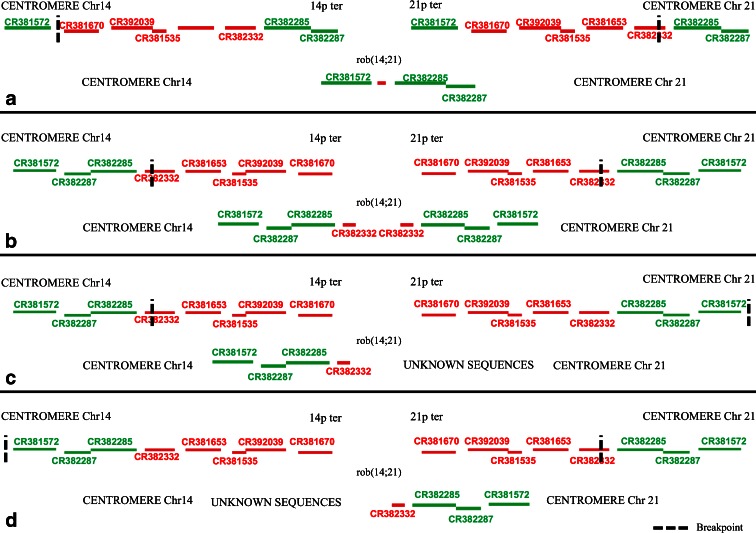
Hypothetical breakpoints (*the dotted line*) in rob(14;21), clones marked in *green* are present (based on FISH results) in mcROBs, while clones marked in *red* are absent. The breakpoints according to Lyle results and the hypothesis about opposite orientation of sequences on 14p than on 21p (**a**). Our PCR and FISH results indicate that the breakpoints are in/nearby to clone CR382332 in both (**b**) or only in one (**c**–**d**) chromosome involved in rob(14;21)

Based on our results showing the presence of these eight clones also on p arm of chromosomes 13, 14, and 21, as well as the hypothesis that the sequences on 14p are arranged in the opposite orientation than the homologous sequences present on 13p and 21p, the breakpoint could be placed between the fragment of CR382332 and clone CR381572 (Fig. 5a). In order to verify the above hypothesis, we conducted interphase FISH on monochromosomal hybrids contained chromosome 13, 14, or 21. This analysis determined the orientation of analyzed clones, in relation to the centromere. Because of the highly repetitive sequences of the most analyzed clones and occurrence of clone CR382287 fragment in hamster genome, we selected 3 BAC clones, namely CR382285, CR381572, and CR382332. These clones were hybridized to interphases in the presence of alpha satellite probes, specific to centromere region of chromosomes involved in mcROBs in two combinations (1) CR381572, CR382332, and alpha satellite probes and (2) CR381572, CR382285, and alpha satellite probes, but only FISH using CR381572 and CR382332 gave the unambiguous results. For all analyzed chromosomes, we observed the following probes order: centromere—CR381572—CR382332—telomere, whereas for clones CR381572 and CR382285, we noticed a lot of cells with undetermined order. According to these results, we can assume that the hypothesis is incorrect. Another aspect of our results is that they indicate different orientation and order of analyzed clones than presented by Lyle et al. (2007) and hg19/GRCh37 human Genome Browser. First of all, it means that clone CR381572 is localized proximal to centromere of chromosome 14 and 21, whereas CR382332 is distal (Fig. 4). Admittedly, we are not able to align all eight clones, but our results demonstrate that the group of four clones CR381535, CR381653, CR381670, and CR392039 are distal to centromere and absent in mcROBs, while the clones CR382285, CR382287, and CR381572 are proximal to the centromeres of investigated chromosomes and they are present in mcROB. For the reason that fragment of clone CR382332 was amplified in PCR whereas no FISH signal was detected, we presume that the region of breakpoint could be placed in or nearby to clone CR382332 in both (Fig. 5b) or only in one (Fig. 5c–d) chromosome involved in mcROB.

Determination of the breakpoint exact location in the rob(13;14) and rob(14;21) remains the difficult task because of extensive variation size of polymorphic sequences in the p arm of acrocentric chromosomes and the lack of mapping data of these regions. Although the Human Genome Project covers only euchromatin regions, we were able to localize these translocations’ breakpoint in the same sequence fragment in all studied mcROB. Moreover, our study using eight clones, identified by Lyle, aiming for narrowing the breakpoints in mcROB, is the first after these sequences appeared in databases. The presented results provide additionally the most precise accessible information about sequences in short arms of acrocentric chromosomes and possible breakpoint region in mcROB which will be helpful both in further studies and in understanding the mechanism of their formation.

## Electronic supplementary material

Below is the link to the electronic supplementary material.

ESM 1 (PDF 170 kb)

## Abbreviations

BAC: Bacterial artificial chromosome
FISH: Fluorescent in situ hybridization
ROB: Robertsonian translocation
mcROBs: Most common Robertsonian translocations
