# Supplementary material for: Narrowing the localization of the region breakpoint in most frequent Robertsonian translocations
Source: Chromosome Res. 2014 Sep 2;22(4):517–32. doi: 10.1007/s10577-014-9439-3 (PMC4257996; doi:10.1007/s10577-014-9439-3)
Supplement: Supplementary file 1 — (PDF 170 kb) [file 10577_2014_9439_MOESM1_ESM.pdf]

Narrowing the localization of the region breakpoint in most frequent Robertsonian translocations.

Chromosome Research

Malgorzata Jarmuz-Szymczak,[1,2,4,*] Joanna Janiszewska,[1,4] Krzysztof Szyfter,[1] Lisa G. Shaffer[3]

1. Institute of Human Genetics, Polish Academy of Sciences Poznan, 60-479 Poland
2. Department of Hematology, Poznan University of Medical Sciences, 60-569 Poznan,
3. Paw Print Genetics, Genetic Veterinary Sciences, Inc., Spokane, WA 99202 USA
4. These authors have contributed equally to the manuscript.

* Corresponding author:

e-mail: maljar@man.poznan.pl

Table 1. Characterization of BAC clones. * Working Draft, ** clone contains a fragment of mouse sequence.

BAC clones				
GRCh37/hg19	NCBI36/hg18	Library	# chr.	bp
GL000149	CR382285	bP-21201H5	21	178 865
GL000149	CR382287	bP-21216K13	21	278 310**
GL000148	CR381653	bP-21264C1	21	131 056
GL000147	CR381535	bP-2154M18	21	152 296
GL000147	CR392039	bP-2171C21	21	158 069
GL000146	CR381670	bP-2189O9	21	129 889
GL000145	CR381572	bP-2168N6	21	184 355
absent	CR382332*	bP-21120F14	21	166 452

Table S2. Characterization of cell lines and culture conditions.

Cell lines	Chromosome	Sample type	Cultured media	%CO_2
**Monochromosomal cell lines**				
GM 10898D	13	human-hamster	DMEM + Prolina	10
GM 10479B<br>GM 11535A<br>CP43	14	human-mouse<br>human-hamster human-hamster	F12+G418<br>RPMI-1640<br>HAM'S	5<br>5<br>5
GM 11715A	15	human-mouse	MEM+ G418	5
GM 10323A<br>GM08854	21	human-mouse<br>human-mouse	MEM<br>DMEM	5<br>10
GM 10888A	22	human-hamster	DMEM	10
GM 63171A	Y	human-hamster	MEM	5
GM 10115B	4	human-hamster	DMEM +Prolina	10
GM 10611	9	human-hamster	RPMI + Histydynol	5
GM 13139A	1	human-mouse	MEM+ G418	5
**Somatic hybrids**				
1	rob(14;21)	human-hamster	DMEM + HAT	10
2	rob(14;21), 4	human-hamster	DMEM + HAT	10
3	rob(14;21), Y	human-hamster	DMEM + HAT	10
4	rob(14;21), 9	human-hamster	DMEM + HAT	10
1Ma	14,14,21	human-hamster	DMEM + HAT	10
1Mb	21	human-hamster	DMEM + HAT	10
5	rob(14;21), 9q	human-mouse	DMEM + HAT	10
8	rob(13;14), 13	human-hamster	DMEM + HAT	10
**Lymfoblastoid cell lines**				
2-M	control	Lymphoblast	RPMI	5
5-M	control	Lymphoblast	RPMI	5
6	rob(14;21)	Lymphoblast	RPMI	5
6-M	control	Lymphoblast	RPMI	5
7-M	control	Lymphoblast	RPMI	5
8-M	control	Lymphoblast	RPMI	5
9	rob(13;14)	Lymphoblast	RPMI	5
9-M	control	Lymphoblast	RPMI	5
10	rob(13;14)	Lymphoblast	RPMI	5
10-M	control	Lymphoblast	RPMI	5
11	rob(13;14)	Lymphoblast	RPMI	5
12	rob(13;14)	Lymphoblast	RPMI	5
13	rob(13;14)	Lymphoblast	RPMI	5
14	rob(13;14)	Lymphoblast	RPMI	5
15	rob(13;14)	Lymphoblast	RPMI	5
16	rob(13;14)	Lymphoblast	RPMI	5
17	rob(13;14)	Lymphoblast	RPMI	5
18	rob(13;14)	Lymphoblast	RPMI	5
19	rob(13;14)	Lymphoblast	RPMI	5
20	rob(13;14)	Lymphoblast	RPMI	5
21	control	Lymphoblast	RPMI	5
22	control	Lymphoblast	RPMI	5
**Controls cell lines**				
3 males	controls	Lymphoblast	RPMI	5
3 females	controls	Lymphoblast	RPMI	5
A9 cell line (mouse)	control for PCR	Fibroblast	DMEM	10
RJK88 (hamster)	control for PCR	Fibroblast	DMEM	10

Table S3. The list of primers used for PCR reactions.

Name	Nucleotide sequences	Length of products	Annealing temp. ºC
381653-1F	GCTACCAAATCCCTTCCTCA	199	58
381653-1R	CCTATCCTTGCCATGCTCAC		
381653-2F	GCCCACCTACAAACGTCACT	254	58
381653-2R	AGGTTTCATCCATCCATCCA		
381653-1aF	gaggtgtggagtgatgtgga	247	60
381653-1aR	ccctacACCCTTctccactg		
381653-2aF	gagttgagtggagggacgag	227	58
381653-2aR	ctggagtgcagtggcaca		
381653-3F	gatagtgccactgcactcca	217	58
381653-3R	ccatcccattgcattttacc		
381653-5F	tttccaactgagcctccact	209	58
381653-5R	tttcagcatttttgcactgg		
381653-7F	ACTGGTGCTTCAGAGGAGGA	227	58
381653-7R	ACCCCAGACCACACTTTCAG		
381653-8F	agcatctctcctgccacact	218	58
381653-8R	aaaaccattctggggctctt		
381653-10F	GCATTGCCCTTTCATCTAGC	242	58
381653-10R	TGATCTTATGGCGGAGAAGG		
382285-1F	CCTTGAGCCAAAATTGAGGT	2018	60
382285-1R	TTGGCCATGAGCATTCCTAT		
382285-2F	AATGGAATTCCTGGGTCACA	2143	60
382285-2R	TTTTGCTTTCTTCCCTTCTTG		
382285-3F	CACGGATTCGTCTTCTCCAT	167	59
382285-3R	TGCCTCCCATCATCTAGCTC		
382285-4F	TGGCTATCTGATCGAAAAACAA	182	59
382285-4R	TCAAACTCGTATCCCTGCAA		
382285-5F	CAGAGCAGATTGGGAAGGAA	249	58
382285-5R	GAGCCCCATAAATCCACAAC		
382285-6F	TCAACCCTATCTCGCCACTT	176	59
382285-6R	GTGTCCCTACTCCCTGATGC		
382285-7F	TAGGAGCTCAGCACAAAGCA	320	59
382285-7R	GGTGCCTTCTTCCCATGTTA		
381535-1F	GGGTCCTGTTTGACAGCAAT	254	59
381535-1R	TGGCTTCTTTTCCCCTACCT		
381535-2F	CCCTACACAGGATTCCCAGA	201	58
381535-2R	GTGTTTCATGGCTCCCATCT		
381535-3F	GTACCACCCGTCAGCAAAAT	191	59
381535-1R	CTTGTGTCTCCACTGCCTCA		
381572-1F	CCCATGTTTCGTCAGGTCTT	2016	60
381572-1R	AAAAATCACGTCGGCAAAAG		
381572-2F	ATAAAGTCGCTTGGGCAAAA	2420	60
381572-2R	TTTGCCAATCTCTGCAGTTG		
381572-3F	TGTATCAGGGCTCAGGGACT	242	59
381572-3R	AGGATGGGGATGCTCTCTCT		
381572-4F	GCCCAGGGCATTCACTACTA	246	59
381572-4R	TTCAACTTCTGGAGCACACG		
381572-5F	GGAATCTGAGCAGCATACCTG	204	58
381572-5R	GACGCTCAAGGCATTTCTTC		
381572-6F	GTCTCTTTGGGCCTTTCCTT	195	59
381572-6R	AACTTGGCCTTTGTGGAGTG		
381572-7F	GCTGAAAGAGCAGGGTCCTA	299	59
381572-7R	AGGCTCCCCTCCTAGAATCA		
381572-8F	CTGAGATCATGACCCAAGAGC	345	59
381572-8R	CTTCCACCCACAAGGAATGT		
381572-9F	GGCAAAATTGAAGTGGCAGT	194	58
381572-9R	TGGTTCTTTCATTGCTGGAA		
381572-10F	GGCAGGATGAGGACACATTT	289	59
381572-10R	TGCTGGCTTATTTCTCCCTA		
381572-11F	AGGCCATCTGTTCCCTTCTT	291	59
381572-11R	TGACAGCGTTTTGGAAGTCA		
381572-12F	AGTAAGGCTGCAGGGTAGCA	201	59
381572-12R	CCAGGGTTGCCAAAAGATAA		
381670-1F	TAACATCCAGTGGGGAGAGG	2240	60
381670-1R	ATATACGGTGGGCCCATACA		
381670-2F	ACCTGAGGCAAGTCAGGAGA	2189	60
381670-2R	CCAACCAAACATTCATGCAG		
381670-3F	ACTGGTGCTTCAGAGGAGGA	227	58
381670-3R	ACCCCAGACCACACTTTCAG		

381670-4F	AAGGAGAAACGGATGTGTGC	205	59
381670-4R	GAAACGGCATGTAGCGATTT		
381670-5F	GCCACCTTTGGGACAATCTA	195	59
381670-5R	agtcagggggaggtcttgtt		
381670-6F	GTCTGAACCTTTGCCTGGAG	204	59
381670-6R	AGGTCAATGCCACTCCATCT		
381670-7F	TGGGATCTCTGGCTAGGTTG	247	58
381670-7R	GTTGGCAGGCATACAGGAGT		
381670-8F	CTGGATGGGAAAACCAGCTA	230	59
381670-8R	GGAGACACCACAAGGCAAAT		
382287-1F	CCCATCATGGAGAGCTGTTT	1663	60
382287-1R	CCTCCAGAAATTTGTTGAAACC		
382287-2F	CCTTAAGGCCAGGCTGAATA	2148	60
382287-2R	TCAGCATTCTGAAAGCACAGA		
382287-3F	AATGCAGAGAGGGTTTGGAG	180	59
382287-3R	GGTGGTTGCTCTTTTCCTGA		
382287-4F	TCCATGGCAAGCACTATATCC	217	59
382287-4R	TGGCAGACCTACCCTCACAT		
382287-5F	TGGGCTCTGCTTTAGCTTGT	185	58
382287-5R	TTTTCTCTTCCCACCACCTG		
382287-6F	CCGTTGACAGCTTCCTTCTC	197	59
382287-6R	AGCAGCCTCTTTGCTAGGC		
382287-7F	TACCCTGTGAACCCATGACA	216	59
382287-7R	ATGGAAATGCTTGCCAACTC		
392039-1F	CTTCTGGTCATGCCAAGGTT	251	59
392039-1R	TTCACACTAGGGCACCTCCT		
392039-2F	CATGGTGGAGATCAGGTGTG	242	58
392039-2R	AACCCCTCCCTCTGTGAACT		
392039-3F	TTTCCCCAATTCCAGTGTTC	242	59
392039-3R	GCCCCTTGAGTGTTCAATGT		
382332-1F	AGGTGGAAACGGATTCTGTG	172	59
382332-1R	AGCCTCCCACTTTAGGCAAT		
382332-2F	GCCTGAGAAGCAATTCGTGT	298	58
382332-2R	AAAAGCAGAGGAGTGGGTTG		
382332-3F	AGAGGACCACAGAAGGCAGA	203	59
382332-3R	CATAGACGAAACGAGCAGCA		
382332F4	AAGGCATCTTGGAAAGGAAC	247	59
382332R4	GGCAGGGAAAGTGCTAAAAA		
382332F5	TTGAGGGAGCACAGTGGTTA	351	54
382332R5	TGCTTTTTGCTGACAAGACA		
382332F6	ATCCAGAATTTGTAGGGCTT	183	58
382332R6	AATCTCCATGGTGTTCGAAC		
382332F7	gcacccaatacaggagcact	248	58
382332R7	gcaatgtggtgctgagaaga		
382332F8	taaagggcagccagagagaa	238	58
382332R8	ggcctggtggtgacaatatc		
382332F9	gggagaaaccagtgcaaaaa	192	58
382332R9	ttgggttagaacacgctcct		
382332F10	ccactgtgttccagcctttt	203	52
3 82332R10	tccaccctaatccactgaca		
3 82332F11	aggttgcagtgagccaagat	150	52
3 82332R11	gtaatgCCCTCTCCctctcc		
3 82332F12	ggaggttgtagcgagcagag	202	63
3 82332R12	cgcctgactggttttcgtat		
3 82332F13	caaagacacaacgtcccaga	169	52,6
3 82332R13	tgctagcttttgaattgtttgttc		
3 82332F14	TCCTGTTGAAAGAAATTAATGGA	176	52
3 82332R14	TCCCATTGCATTTTACCTCA		
3 82332F15	ggaagggaatgggaagga	222	59
3 82332R15	ccactccatttccctcaact		
D21S1276-F	GGCCTGATGTCTGCCTTAGAT	100	60
D21S1276-R	GCCATAGGTGAGCAACAGGA		
D21S188-F	TTCTGTGTCATCTGAACTGG	180	60
D21S188-R	ACGCACATTGAATACTGAGG		
D14S128- F	TGAGGTTATGCATGTGTGAG	326-389	59
D14S128- R	GCCCCAAAACTCTCAGAAG		
D14S139- F	ATGGGAACTGTTCAAGTCTC	231-270	57
D14S139- R	GGTCTTCGTTCCAATAACCC		
D14S283- F	ACTATATCTCCCAGGC	125-153	58
D14S283- R	TGTTTTCCTAGTAACCGCA		
D13S1275-F	ATCACTTGAATAAGAAGCCATTTG	180-214	58
D13S1275-R	CCAGCATGACCTTTACCAG		

D13S162-F	GCAATCTGAAACATTCTCCA	107	58
D13S162-R	CCACATGGAAAATGAAATTG		
D13S175-F	ATGGGAGAAATGGGAAGATGTTGG	150	58
D13S175-R	CACTTAAAATCTACTCTCTCAGCAG		
